# Gastrointestinal Microbiota Do Not Significantly Contribute to T Cell Activation or GI Inflammation in Ndfip1-cKO Mice

**DOI:** 10.1371/journal.pone.0034478

**Published:** 2012-04-10

**Authors:** Vanessa Kurzweil, Amy Tarangelo, Paula M. Oliver

**Affiliations:** 1 Cell and Molecular Biology Group, Perelman School of Medicine at the University of Pennsylvania, Philadelphia, Pennsylvania, United States of America; 2 Department of Biology, University of Pennsylvania, Philadelphia, Pennsylvania, United States of America; 3 Children's Hospital of Philadelphia and Department of Pathology and Lab Medicine, University of Pennsylvania, Philadelphia, Pennsylvania, United States of America; Ulm University, Germany

## Abstract

The bacteria inhabiting the mammalian gastrointestinal (GI) tract play a vital role in normal digestion and immune function. In a healthy host, the immune system is tolerant to gut bacteria and does not mount an effector response to bacteria-derived antigens. Loss of tolerance to intestinal microflora has been associated with inflammatory bowel disease (IBD) in both mice and humans. Mice lacking Ndfip1, an adaptor protein for E3 ubiquitin ligases of the Nedd4-family, in T cells (Ndfip1-cKO) develop a disease resembling IBD. Inflammation in these mice is characterized by increased activation of peripheral T cells, infiltration of eosinophils into the GI tract, and epithelial hypertrophy in the esophagus. We hypothesized that this intestinal inflammation in Ndfip1-cKO mice is caused by a loss of T-cell tolerance to bacterial antigens. Here, we show that treatment of Ndfip1-cKO mice with broad-spectrum antibiotics drastically reduced bacterial load in stool but had little effect on T-cell activation and did not affect eosinophil infiltration into the GI tract or epithelial hypertrophy in the esophagus. Thus, inflammation in Ndfip1-cKO mice is not caused by a loss of tolerance to intestinal microbiota. Rather, T cell activation and eosinophilia may instead be triggered by other environmental antigens.

## Introduction

Bacteria in the mammalian gut play a crucial role in host homeostasis, aid digestion, provide competitive barriers to pathogen invasion, and contribute to immune system development [1]. While the intestinal immune system is capable of mounting a rapid effector response to pathogenic bacteria, it is tolerant toward gastrointestinal microflora and does not normally mount an immune response against bacteria-derived antigens [2]. A loss of tolerance to gastrointestinal bacteria has been implicated in the pathogenesis of many mucosal diseases, including inflammatory bowel disease (IBD) [2]–[5].

It was recently shown that mice deficient in Nedd4-family interacting protein-1 (Ndfip1) develop IBD-like symptoms [3]. Ndfip1 is an adaptor for E3 ubiquitin ligases of the Nedd4-family, including Itch. Itch can promote ubiquitin-mediated degradation of various signaling proteins, thereby suppressing T cell activation and inflammation [4]. Ndfip1 has been shown to promote Itch function in vivo. For example, Ndfip1 promotes Itch ubiquitylation and subsequent degradation of JunB, a transcription factor that increases production of proinflammatory T_H_2 cytokines interleukin (IL)-4 and IL-5. In the absence of Ndfip1, JunB accumulates and promotes T_H_2 cytokine transcription [5]. Mice lacking Ndfip1 develop severe T_H_2-mediated inflammation at sites of environmental antigen exposure, including skin, GI tract, and lung. GI inflammation in Ndfip1−/− mice is preceded by aberrant activation of CD4+ and CD8+ T cells and increased IL-5 production, followed by recruitment of eosinophils into the GI tissue [3]. Transfer of Ndfip1−/− CD4+ T cells is sufficient to induce GI inflammation in Rag1−/− mice [3]. Additionally, mice lacking Ndfip1 only in T cells (referred to here as Ndfip1 cKO mice) develop GI tract symptoms similar to those observed in Ndfip1−/− mice [6]. These observations suggest that, in the absence of Ndfip1, aberrant T-cell responses drive eosinophil recruitment and inflammation in the GI tract.

The localization of inflammation in Ndfip1−/− mice to sites of environmental antigen exposure suggests loss of tolerance to environmental antigens. Thus, we hypothesized that GI inflammation in Ndfip1−/− mice is caused by an inappropriate T-cell response to gastrointestinal microbiota. In several genetic models of IBD, depletion of intestinal bacteria by oral antibiotic treatment greatly reduces intestinal inflammation [7], [8], [9]. We therefore tested the role of bacterial antigens in triggering the Ndip1−/− phenotype by treating Ndfip1 cKO mice with a cocktail of antibiotics and subsequently analyzing tissues for signs of inflammation in the GI tract.

## Materials and Methods

### Mice

Ndfip1-cKO mice have been described [6]. All experiments described in this manuscript compare Ndfip1-cKO mice to littermate controls. These controls include mice with one or two Ndfip1 floxed alleles but not expressing Cre, or mice expressing Cre that do not have either Ndfip1 allele floxed. No differences were observed when comparing these two types of controls. All mice were bred in the Children's Hospital of Philadelphia animal facility. All experimentation was approved and followed guidelines established by the institutional animal care and use committee of the Children's Hospital of Philadelphia.

### Genotyping

Ndfip1-cKO mice were genotyped using purified genomic DNA from tail samples and the following PCR primers: Ndfip1 floxed forward 5′-TGAGGAAACAGACACACAATG-3′, Ndfip1 floxed reverse 5′- TGGAATGAACCTGAGGTCTCC-3′. Samples were amplified using conventional PCR techniques and run by electrophoresis on a 1% agarose gel. Wild type DNA forms an approximately 1 kb band and Ndfip1−/− DNA yields an approximately 340 bp band.

### Antibiotics

Animals were provided with autoclaved drinking water supplemented with sucralose (1.5 mgmL^−1^) or autoclaved drinking water supplemented with sucralose (1.5 mgml^−1^), ampicillin (0.5 mgml^−1^), gentamicin (0.5 mgml^−1^), metronidazole (0.5 mgml^−1^), neomycin (0.5 mgml^−1^) and vancomycin (0.5 mgml^−1^). For mice treated upon weaning, animals were 26 days old at the start of treatment and were sacrificed 14 days later. For mice treated since birth, pregnant females were treated starting approximately 1 day before giving birth until their litters were weaned, at which point weaned pups were treated for a further 14 days and sacrificed.

### Lymphocyte isolation from tissues

At necropsy, mesenteric lymph nodes (mLN), spleen, esophagus and small bowel were harvested. Single-cell suspensions of spleen and mLN were prepared by mashing tissue through a 70 µm filter. LNs were then washed and resuspended in phosphate-buffered saline (PBS). Splenic red blood cells were lysed with ACK buffer (.15 M Nh_4_Cl, 10 mM KHCO_3_, .1 mM EDTA) and samples were then washed and resuspended in PBS.

For preparation of lymphocytes from the small bowel, a 3–4 inch section of small bowel was removed. Peyer's patches were excised and the luminal contents were removed.

Small bowel and esophagus samples were minced and then digested in medium containing collagenase type 1, collagenase type 1a, and DNAse for approximately one hour at room temperature. Samples were then strained through a 100 µm filter, washed with Dulbecco's Modified Eagle Media (DMEM), and filtered again through a 40 µm filter.

### Flow Cytometry

Single-cell suspensions of lymphocytes isolated from tissues were stained for surface expression using fluorochrome conjugated anti-CD3, anti-CD8α, anti-CD8β, anti-CD4, anti-F4/80, anti-CD62L, anti-CD44, and anti-Siglec-F (anti-Siglec-F was purchased from BD Biosciences; all other antibodies were purchased from Biolegend). Cells were stained for 30 minutes on ice and washed twice with PBS containing fetal calf serum and sodium azide (FACS buffer). Flow cytometry was performed on an LSRFortessa flow cytometer (BD Biosciences) and results were analyzed using Flow-Jo software (Treestar USA).

### Histological Analysis

At necropsy, small bowel and esophagus sections were removed, fixed in 10% formalin for 48 hours, and embedded in paraffin. Sections were stained with eosin and hematoxyin. Stained sections were analyzed using a Leica microscope (Bannockburn, IL) with a bright field objective.

### DNA Extraction

Stool samples were collected on day 0 of the experiment prior to sucralose or antibiotic administration and on day 13 (for mice treated upon weaning) or day 34 (for mice treated since birth). Samples were stored at −80 C and later defrosted and lysed by bead beating using the Mini BeadBeater-16 (Biospec). The samples were then prepared using the QiaAmp DNA Stool Mini Kit (Qiagen) according to the manufacturer's instructions. Final DNA concentrations were measured using a Thermo Nanodrop 2000.

### Bacterial quantitation by 16S qPCR

16S quantitation was performed using real-time PCR of 20 uL triplicate reactions containing 10 ng stool DNA, TaqMan Environmental Master Mix 2.0 (Applied Biosystems), and primers/probe specific for bacterial 16S rDNA as described by Hill, *et al.* [10]. Standard curves with a range of 10^1^ to 2×10^7^ copies of *E. coli* 16S rDNA were prepared using linearized plasmid containing a single copy of the 16S gene.

## Results

### Ndfip1-cKO mice Display T-cell Activation and GI Inflammation

Ndfip1-deficient mice develop a severe inflammatory disease by 6 weeks of age. However, the precise age of disease onset in mice lacking Ndfip1 only in T lineage cells was unknown. We sought to characterize Ndfip1-cKO mice at 3 and 5 weeks of age by testing for T cell activation in the spleen as well as T cell and eosinophil infiltration of the small bowel and esophagus.

**Figure 1:**
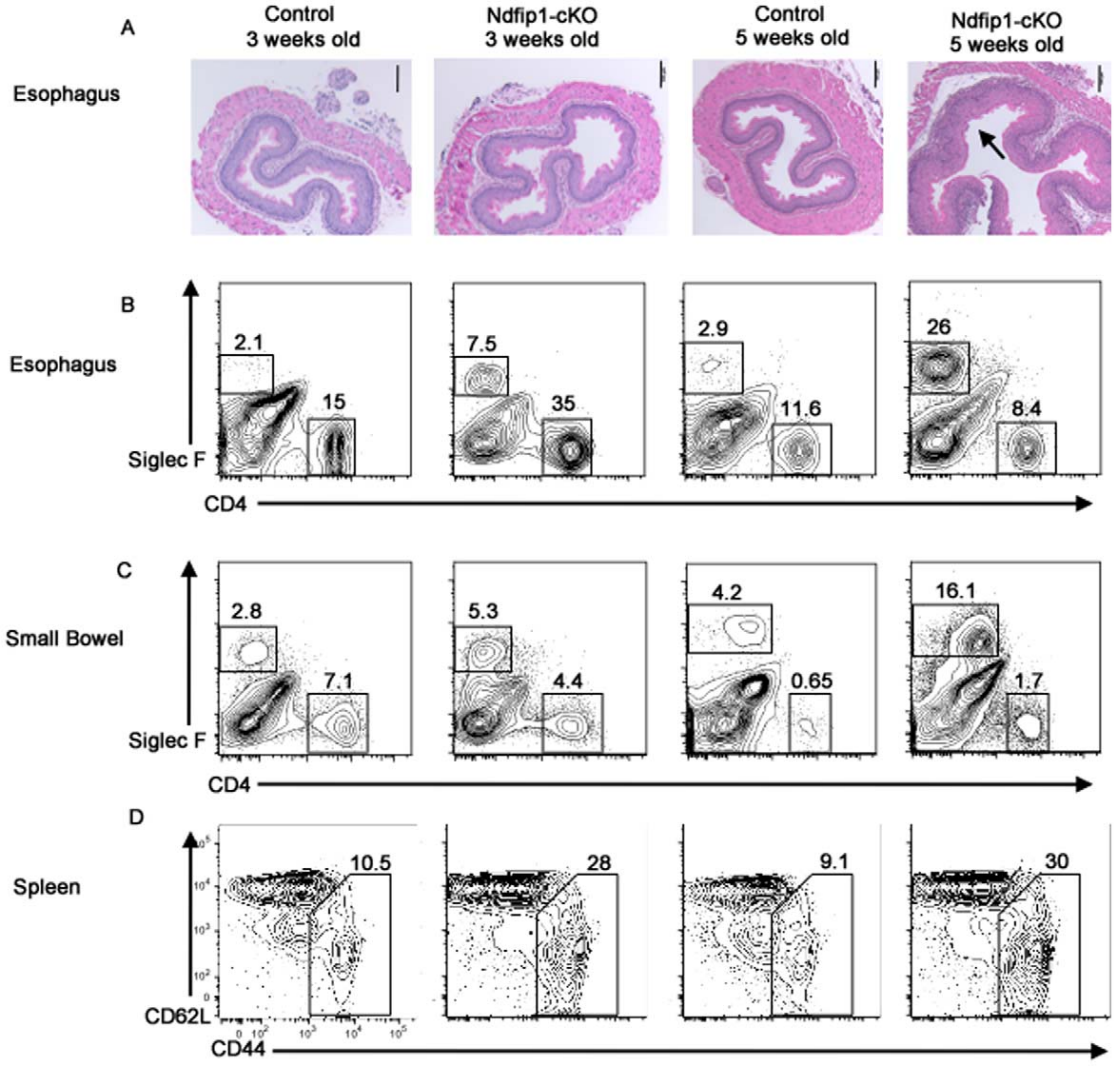
Ndfip1-cKO mice show increased inflammation and eosinophilia between 3 and 5 weeks of age. (A–D) Ndfip1-cKO and control mice were analyzed at 3 weeks and 5 weeks of age for signs of inflammation. Representative histology or flow plots from each group are shown. (A) Histological sections of esophagus were taken from Ndfip1-cKO and control mice at 3 weeks and 5 weeks of age, were stained with H & E and analyzed by microscopy using a 10× objective. (B–C) Representative flow cytometric analysis of cells isolated from the esophagus (B) and small bowel (C). Plots show CD45+, live gated cells. Eosinophils (Siglec F+ cells) and CD4+ T cells are shown. Percentages of populations in the gated regions are indicated. Representative flow cytometric analysis of splenic CD4+ T cells (D). Gate on each plot represents cells defined as activated based on expression of CD44 and loss of CD62L. Percentage of cells in each gate is indicated.

Histological sections of esophagus showed increased inflammation and hypertrophy of the esophageal epithelium of Ndfip1-cKO mice at 5 weeks of age. However, no discernible difference between control and Ndfip1-cKO mice was observed at 3 weeks (Figure 1A). Flow cytometric analysis of cells isolated from the esophagus showed an increased percentage of Siglec F+ cells in Ndfip1-cKO mice by 5 weeks of age. In contrast, the percentage of eosinophils in the esophagus of 3 week old Ndfip1-cKO mice was not as profound (Figure 1B). Similarly, an increase in the percentages of eosinophils in the small bowel of Ndfip1 cKOs was observed at 5 weeks, but this was not as evident at 3 weeks-of-age (Figure 1C). Spleens of Ndfip1 cKO mice are typically enlarged 2–3 fold compared with wild-type littermates. Average total spleen cellularity was 101×10^6^ cells for 3-week-old Ndfip1 cKO animals compared to 40×10^6^ for 3 –week-old controls. By five weeks of age the total numbers of cells isolated from the spleens of Ndfip1-cKO mice had increased further, to an average of 124×10^6^. Because of this increase in cellularity, we compared differences in activation of splenic T cells in 3 and 5 week old mice. As shown previously, T cells lacking Ndfip1 are more likely to have a previously activated phenotype, characterized by increased levels of CD44, than control T cells. Interestingly, flow cytometry analysis showed a similar increase in CD4+ T cell activation at both 3 and 5 weeks of age (Figure 1D). Similar results were observed in CD8+ T cells isolated from the spleen (data not shown).

We have shown previously that in Ndfip1−/− Rag1−/− mice that contain T cells specific for an ovalbumin peptide that is not expressed in mice (OTII), T cells maintain a naïve phenotype [6]. Thus, T cells lacking Ndfip1 must be exposed to antigen(s) to acquire an activated phenotype. Taken together, these data suggest that T cells lacking Ndfip1 become activated by antigens and then migrate into the GI tract where they drive inflammation. Knowing that bacterial-derived antigens are a major source of antigens in the GI tract, we hypothesized that these antigens might promote the activation of T cells lacking Ndfip1 and thus drive GI inflammation in the GI tract.

### Antibiotic Treatment of WT Mice Reduces Intestinal Bacteria and Does Not Significantly Alter T cell Activation or GI Eosinophilia

**Figure 2:**
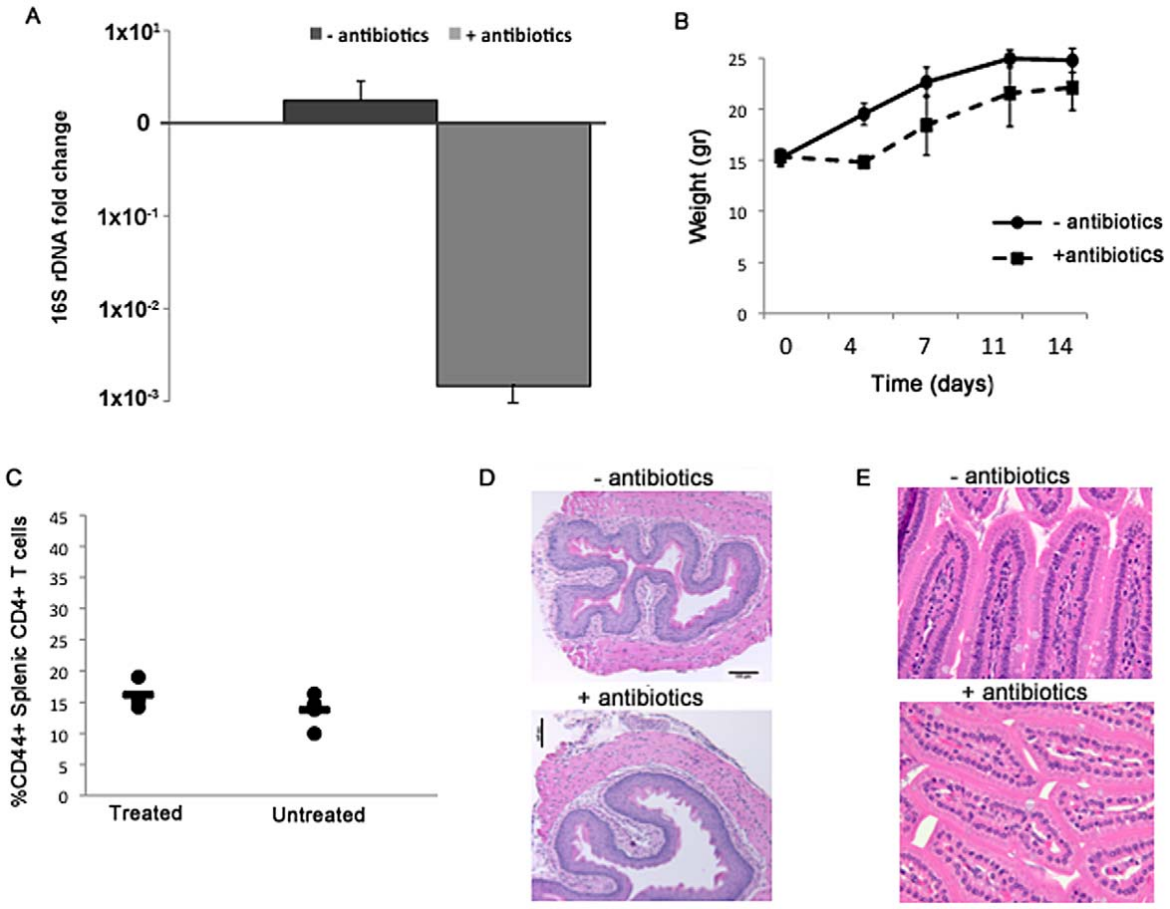
Antibiotic treatment of wild type mice decreases bacterial load and does not produce overt immunological changes. (A) The average fold change in 16 s rDNA copies was quantified by qPCR from stool of wildtype mice treated with antibiotics and sucralose (n=5) or sucralose alone (n=3). Data shown is the fold change in 16S copies after 13 days of treatment. Error bars represent the SD. (B) The weight of mice treated with sucralose only (black line) or sucralose and antibiotics (dotted line) is shown over the course of treatment. Dot represents the mean of the population and error bars are the SD from the mean. (C) Percentages of activated splenic CD4+ T cells (defined as shown in Figure 1D) in mice treated with sucralose only or sucralose and antibiotics is shown. Each dot represents a single mouse. (D–E) Histological sections of the esophagus (D) and small bowel (E) are shown for mice treated with sucralose only (−antibiotics) or sucralose and antibiotics (+antibiotics). A 10× objective was used for esophagus and a 40× objective was used to analyze small bowel.

While oral administration of a broad-spectrum cocktail of antibiotics in drinking water has been shown to be extremely effective in reducing intestinal bacteria, the unpleasant taste can cause mice to avoid drinking and lead to insufficient hydration in treated mice [10]. To ensure effective knockdown of bacteria without causing dehydration, we modified the antibiotic regimen in Hill *et al.* [10] by reducing the concentration of antibiotics and adding sucralose, a non-nutritive sweetener, to encourage drinking. Control cages received drinking water with sucralose only. As shown in Figure 2A, after 13 days this treatment regimen reduced the amount of bacterial 16S rDNA detected in total stool DNA by over 100-fold. In addition, although the mice receiving antibiotics showed an initial loss of weight compared to mice receiving only sucralose, they gained weight steadily over the course of treatment and did not display scruffy coat, lethargy, or other signs of dehydration (Figure 2B and data not shown).

Following treatment, we analyzed wild type mice for signs of immunological changes caused by antibiotic treatment. Flow cytometric analysis revealed no difference in the activation of splenic CD4+ or CD8+ T cells (Figure 2C and data not shown). Additionally, sections of the esophagus and small bowel did not show histological changes following antibiotic treatment (Figure 2D–E).

### Antibiotic Treatment of Ndfip1-cKO mice from 3–5 Weeks of Age Does Not Reduce Inflammation

**Figure 3:**
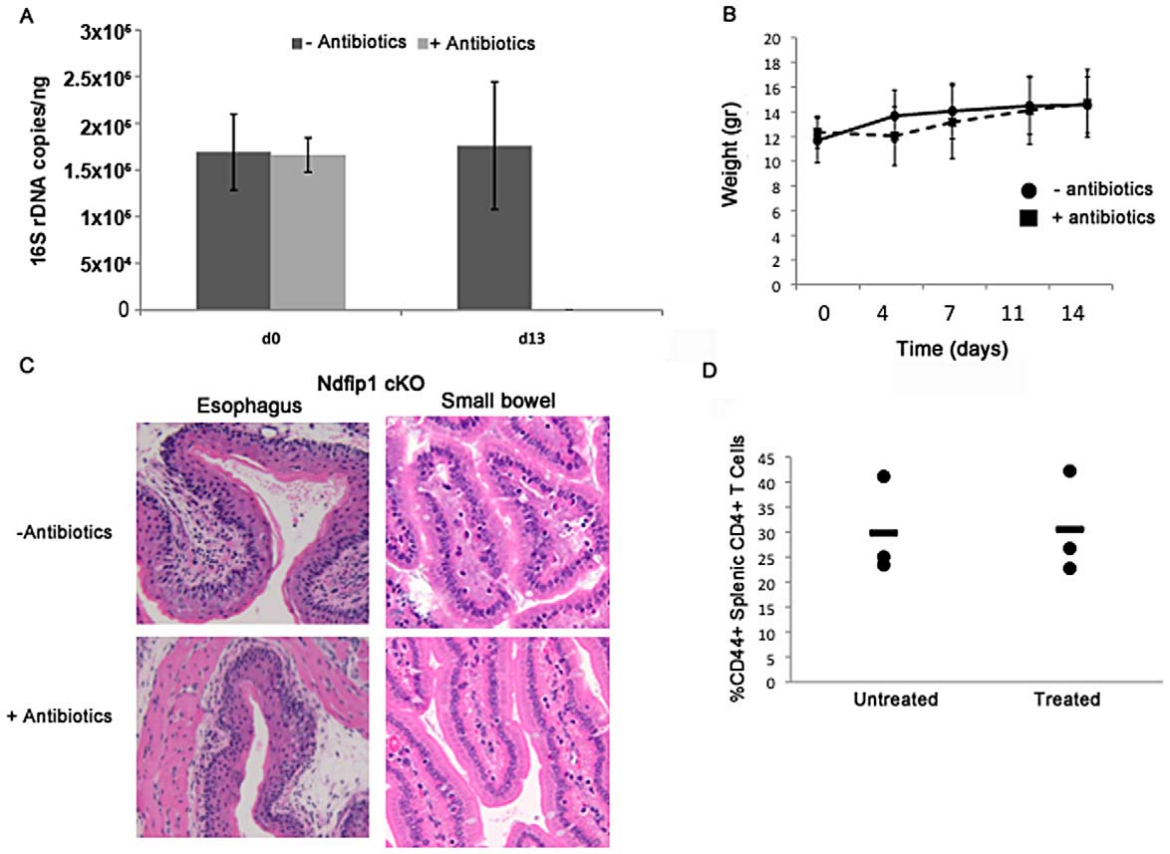
Ndfip1 CD4-cKO mice treated with antibiotics for 2 weeks do not show decreased eosinophilia or reduced inflammation in the esophagus. (A) The average 16s rDNA copies/nanogram of total stool DNA was quantified by qPCR at day 0 and day 13 of treatment with sucralose alone (n=5) or sucralose and antibiotics (n=4). Error bars illustrate SD. (B) Weights of mice treated with sucralose alone (−ABX=solid line) or antibiotics and sucralose (+ABX=dashed line) over the course of the 13 day treatment. (C) Representative H & E stained histological sections of the esophagus and small bowel of Ndfip1-cKO mice after 13 days of treatment with sucralose alone or antibiotics and sucralose are shown. (D) Percentages of activated CD4+ T cells in the spleens of mice treated with sucralose only (untreated) or with antibiotics and sucralose (treated) as determined by flow cytometric analysis. Each dot represents a single mouse.

We next treated age-matched pairs of Ndfip1-cKO and control mice with oral antibiotics as described above. Similar to the results seen in WT mice, antibiotic treatment was extremely effective in reducing bacterial DNA detected in stool and did not cause weight loss (Figure 3A–B). Furthermore, no significant differences were seen in the total amounts of bacteria, as determined by comparing the concentration of 16 s rDNA between Ndfip1-cKO mice and controls, between the two genotypes either before or after antibiotic treatment (data not shown). Despite drastically reducing bacterial flora, antibiotic treatment of Ndfip1-cKO animals did not produce any discernible decrease in inflammation of the esophagus or small bowel. For example, we did not observe a decrease in the hypertrophy in the epithelial esophagus or the number of visible eosinophils infiltrating into the villi of the small bowel following treatment with antibiotics (Figure 3C). Furthermore, flow cytometry revealed no significant change in the percentages of activated CD4+ T cells isolated from the spleen or mesenteric lymph nodes following antibiotic treatment (Figure 3D and data not shown).

### Antibiotic Treatment of Ndfip1-cKO Mice from Birth to 5 Weeks of Age Does Not Reduce T cell activation or GI Tract Inflammation

**Figure 4:**
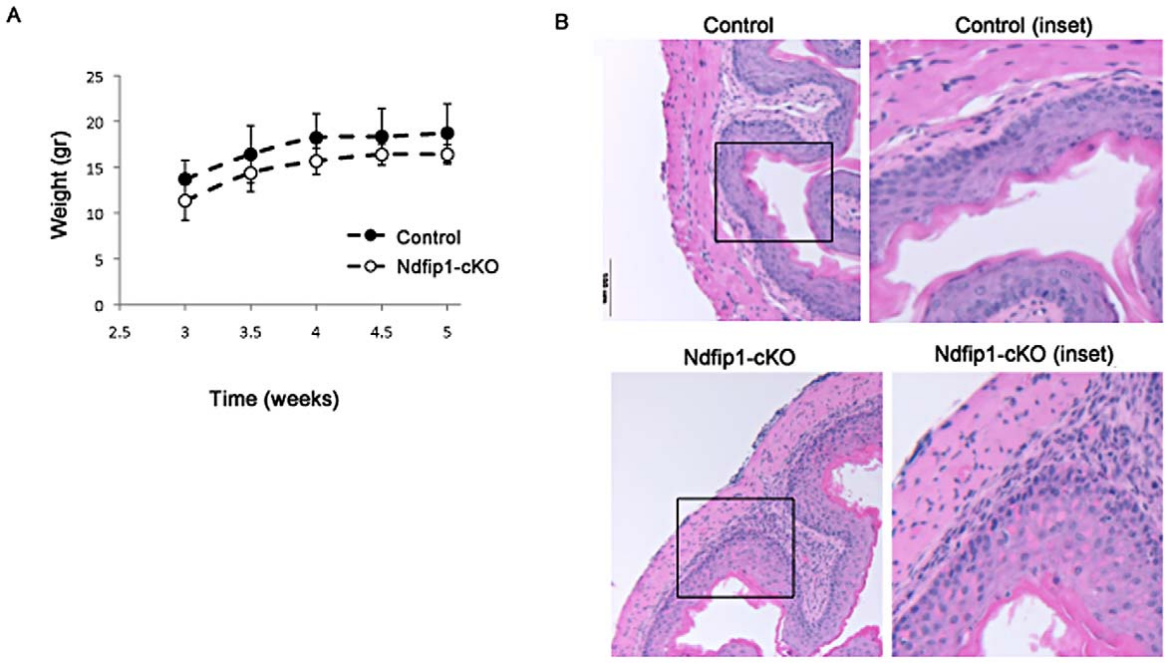
Ndfip1 CD4-cKO mice treated with antibiotics from birth do not show changes in eosinophilia or inflammation in the esophagus, or splenic T cell activation. (A) Weights of control mice (n=4) (closed circles) or Ndfip1-cKO mice(n=4) (open circles) between weeks 3 to 5 of antibiotic treatment. (B) H & E stains of histological sections of esophagus taken from control and Ndfip1-cKO mice after antibiotic treatment from birth to 5 weeks. Images were taken using a 20× objective. Inset of panel outlined by the box is shown in the images on the right.

Because Ndfip1-cKO mice begin to show signs of T cell activation and eosinophilic infiltration into the GI tract as early as three weeks of age, we wondered whether administration of antibiotics beginning at 3 weeks was too late. We hypothesized that treatment from birth would be more effective in reducing pathology. To test this, we administered broad-spectrum antibiotics to pregnant females beginning 1 day prior to their expected delivery date as described above. Treatment continued until mice were analyzed at 5 weeks of age. Antibiotic treatment of Ndfip1 cKO animals using this regimen resulted in similar levels of 16S rDNA in stool at 5 weeks of age as when treatment was administered from 3–5 weeks of age (data not shown). Importantly, mice treated with antibiotics gained weight steadily over the course of treatment (Figure 4A).

**Figure 5:**
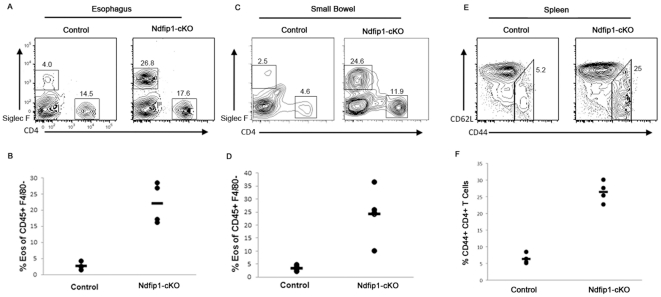
Antibiotic Treatment of Ndfip1 cKOs from Birth Does Not Reduce Inflammation. (A–D) Representative flow cytometry plots of cells isolated from the esophagus (A) and small bowel (C) of Ndfip1-cKO and control animals treated from birth to 5 weeks. Graphs of the percentages of eosinophils (Siglec F+) and CD4+ T cells in esophagus (B) or small bowel (D) from all mice in the experiment are shown. (E) Representative flow plots illustrating the percentage of activiated cells among splenic CD4+ gated T cells. (F) Percentages of activated T cells in the spleens of all mice treated with antibiotics from birth to 5 weeks are shown.

Similar to our observations of mice treated from 3–5 weeks of age, we did not observe any visible reduction in inflammation following antibiotic treatment from birth. The esophageal epithelium of Ndfip1 cKO mice treated from birth was inflamed compared with age-matched controls (Figure 4B). Flow cytometric analysis of cells isolated from the esophagus and small bowel revealed increased percentages of eosinophils in Ndfip1-cKO compared with controls (Figure 5A–D). The representative plot in figure 5C shows increased percentages of (Siglec F+) eosinophls among cells isolated from the esophagus (Figure 5A) or small bowel (Figure 5C) of an Ndfip1-cKO mouse. Furthermore, elevated percentages of eosinophils in these tissues were observed in all Ndfip1-cKO animals under investigation (Figure 5B and D). Additionally, all Ndfip1-cKO mice examined demonstrated a substantial increase in the percentages of activated T cells in their spleens after this course of antibiotic treatment (Figure 5E and F). The percentages observed were similar to those percentages in untreated 5 week old Ndfip1-cKO mice. Thus, we do not see any decrease in the inflammatory phenotype of Ndfip1-cKO mice treated with antibiotics from birth until five weeks of age. Therefore, starting the antibiotic treatment at birth and treating mice until 5 weeks of age did not improve T cell activation or inflammation in Ndfip1-cKO mice.

## Discussion

In this study, we found that treatment of Ndfip1-cKO mice with oral antibiotics did not significantly reduce T cell activation or gastrointestinal eosinophilia and inflammation. No discernible decrease in T-cell activation or eosinophil infiltration was seen in Ndfip1-cKO mice regardless of whether they were treated with antibiotics for two weeks, from 3–5 weeks of age, or treated from birth to 5 weeks of age. This was true despite the efficacy of antibiotic treatment in reducing the total GI bacterial load by more than 100 fold. These results suggest that gut microbiota do not play a significant role in the development of GI inflammation in Ndfip1-cKO mice.

Microbiota are known to influence many aspects of immune function [10], [11], [12]. In a study using oral gavage to administer a similar regimen of antibiotics to WT mice, Hill *et al*. [13] demonstrated that a ten-fold reduction of 16S rDNA in stool. This decrease in GI microbiota correlated with reduced production of IL-17, a cytokine associated with specific types of bacteria [14], [15], [16], by intestinal CD4+ T cells. Our antibiotic treatment reduced the number of bacterial 16S rDNA copies detected in stool by over 100-fold after two weeks. Given the drastic reduction in total bacterial load after antibiotic treatment, we consider it unlikely that any remaining bacteria would provide sufficient antigenic stimulation of the immune system to induce inflammation in Ndfip1 cKO mice. While it remains possible that some bacteria are resistant to the antibiotic treatment and remain in the gut, the ∼100 fold decrease in bacterial load did not correlate with any discernable decrease in the percentages of activated T cells in Ndfip1-cKO mice. This leads us to conclude that few, if any, Ndfip1−/− T cells are activated by bacterial antigens. Thus, the activation of T cells lacking Ndfip1 must be caused by other self- or environmental-antigens.

Our study shows that the gastrointestinal microbiota do not play a significant role in the development of GI inflammation in Ndfip1-cKO animals, it is possible that other types of environmental antigens are responsible for inflammation in Ndfip1-deficient mice. In addition to bacterial-derived antigens, food antigens have also been implicated in the pathogenesis of IBD [11]. Supporting this, food restriction diets have been shown to improve IBD symptoms in human patients and in animal models [12], [13], [14].

Food antigens are also associated with other eosinophilic GI disorders. Eosinophilic gastrointestinal disorders (EGID) are chronic diseases characterized by increased numbers of eosinophils in the digestive tract. These disorders, all of which are associated with food allergy, include eosinophilic esophagitis (EoE), eosinophilic gastritis (EG) and gastroenteritis (EGE), and eosinophilic colitis (EC). Because of its association with food allergy, diet has been used to treat patients with EoE [15]–[16]. The high degree of esophageal inflammation, revealed by eosinophilia and epithelial hypertrophy, in Ndfip1-cKO animals bears a strong resemblance to the pathology of EoE and EGE. Thus, Ndfip1-cKO mice may serve as a model for these disorders. This is of particular significance since patients with EoE commonly have other allergic diseases such as asthma and atopic dermatitis. Mice lacking Ndfip1 also develop atopic inflammation in the skin and lung. This might suggest that, like with patients with EoE, eosinophilia and GI inflammation in Ndfip1−/− mice is caused by aberrant immune responses to food antigens. Experiments are currently underway in our lab to test this hypothesis.
